# The Impact of Parent-Child Relationships on Mental Health in Adulthood

**DOI:** 10.1192/j.eurpsy.2025.2053

**Published:** 2025-08-26

**Authors:** N. Kissa, A. Korchi, N. Ait Bensaid, H. El Majdoub, A. Ouanass

**Affiliations:** 1 University psychiatric hospital Arrazi; 2 University Psychiatric Hospital Arrazi of Salé Faculty of Medicine and Pharmacy – Mohammed V University of Rabat, Salé, Morocco

## Abstract

**Introduction:**

Parent-child relationships significantly influence psychological and social development, with lasting effects on mental health. Warm and supportive relationships are linked to higher emotional well-being, while neglectful or strict parenting increases the risk of mental disorders [1]. Even non-abusive but conflictual relationships can raise the risk of psychological issues in adulthood [2][3], and stable family bonds contribute to resilience in facing life challenges [4][5].

**Objectives:**

This study aims to explore and assess the impact of parent-child relationships on mental well-being in adulthood.

**Methods:**

This study uses a Google Forms questionnaire with six sections to assess the impact of parent-child relationships on mental well-being in adulthood. Univariate analysis was conducted in Excel, and multivariate analysis, including a logistic regression model, was performed in R Studio to measure this impact.

**Results:**

Approximately 65% of participants who reported a warm parental relationship exhibit high mental well-being in adulthood. In contrast, 45% of those who experienced frequent parental conflicts report symptoms of psychological disorders. Among participants who experienced neglect in their parental relationships, 55% developed signs of anxiety in adulthood, while 40% show increased resilience to challenges when family relationships during childhood were stable and secure

**Conclusions:**

The parent-child relationship plays a crucial role in mental health in adulthood. Warm and stable relationships are associated with increased emotional well-being and better resilience in facing challenges. In contrast, relationships marked by neglect or conflict increase the risk of long-term psychological disorders. These findings highlight the importance of the family environment for psychological development and the prevention of mental health issues.

**Disclosure of Interest:**

None Declared

